# Simultaneous Replacement of Maxillary Central Incisors with CEREC Biogeneric Reference Technique: A Case Report

**DOI:** 10.5681/joddd.2013.020

**Published:** 2013-05-30

**Authors:** Gokhan Akgungor, Deniz Sen, Eray Bal, Mutlu Özcan

**Affiliations:** ^1^Professor, University of Istanbul, Faculty of Dentistry, Department of Prosthodontics, Istanbul, Turkey; ^2^Research Assistant, University of Istanbul, Faculty of Dentistry, Department of Prosthodontics, Istanbul, Turkey; ^3^Professor, Department of Endodontics, Marmara University, Faculty of Dentistry, Nişantaşı, Istanbul, Turkey; ^4^University of Zurich, Dental Materials Unit, Center for Dental and Oral Medicine, Clinic for Fixed and Removable Prosthodontics and Dental Materials Science, Zürich, Switzerland

**Keywords:** CAD-CAM, CEREC, crowns, dental porcelain, resin cements

## Abstract

Biogeneric Reference Technique (BRT) of the CEREC 3D v.3.8 software is an effective technique for single anterior ceramic crowns because it provides computer-controlled match of the tooth form to the contralateral tooth. BRT also enables the fabrication of two or more anterior all-ceramic crowns simultaneously. This clinical report demonstrates the clinical application of BRT for designing and milling two central incisors in one appointment using a single optical impression. After completing the virtual design of the first central incisor, it was copied and a mirror image was created. The second central incisor was designed using this replicated image and therefore a computer-controlled symmetry was obtained. The crowns were milled from monolithic feldspathic ceramic blocks and adhesively luted with dual-cured resin cement following dentin conditioning. At the two-year follow-up appointment, the restorations were intact, no adverse effects were noted, and the resultant appearance was highly satisfactory for the patient. A step-by-step protocol is described from design to cementation of these restorations.

## Introduction

The technological advances in the field of computer-aided design and computer-aided manufacturing (CAD/CAM) with digital imaging, software design, and milling have created an equivalent alternative to laboratory-generated indirect ceramic restorations.^1^ The CEREC 3D system (Sirona Dental Systems GmbH, Bensheim, Germany) is a chairside application of CAD/CAM technology for reconstructive dentistry. The system includes an acquisition unit consisting of a portable computer, the design software and an optical imaging system.^2^ The milling chamber with two diamonds, mills the final restoration from prefabricated blocks of either ceramic or polymeric restorative materials.^3^

The use of chairside CAD/CAM systems in dental practice enables the clinician to fabricate all-ceramic crowns in a single visit and provide many advantages for both the patient and dentist. Acquiring optical images of the prepared teeth directly with the intra-oral camera eliminates the need for conventional impression procedures and improves patient comfort.^4^ Single-visit ceramic crowns eliminate the need for provisional restorations, increase durability of adhesion to dental tissues and also reduce postoperative sensitivity.^5^ Potential inaccuracies resulting from laboratory fabrication process were diminished and milling the restoration from optimum controlled ceramic blocks increased the reliability by eliminating the material variation found in lab-fabricated restorations.^6^

The major concerns about chairside CAD-CAM restorations is the accuracy of intraoral digital impressions and the resulting internal and marginal fit discrepancies. However, recent studies demonstrated that digital impression systems allow the fabrication of fixed prosthetic restorations with similar accuracy as conventional impression methods.^7,8^ According to the results of previous studies, marginal gap of the feldspathic crowns fabricated with Cerec 3 chairside system ranged from 53 to 94.4 µm and are in clinically acceptable limits.^9,10^

The key point for fabrication of an esthetic anterior all-ceramic crown is to achieve a high degree of symmetry across the midline.^11^ Conventional CAD software use a dental database containing standardized tooth morphologies from which the shape and form of the all-ceramic crown has been chosen.^12^ However, an automatically generated symmetry to the contralateral tooth is difficult to achieve with the standardized morphologies, and in many cases manual adjustments with the design tools of the software is necessary.^13^ In an attempt to reduce the operator dependence, advanced CAD techniques for crown design has been developed.CEREC 3D v.3.8 software (Sirona Dental Systems GmbH, Bensheim, Germany) have introduced biogeneric reference CAD technique, which is supposed to be useful in achieving bilateral symmetry in anterior crown design.^14^

Biogeneric reference technique (BRT) allows the operator to copy the contralateral tooth and create a mirror image of it on the preparation, permitting symmetrical design through the use of the mirrored twin.^15^ BRT is an effective technique for single anterior ceramic crowns because it provides computer-controlled match of the tooth form to the contralateral tooth.^16^ BRT also enables the fabrication of two or more anterior all-ceramic crowns in one visit using a single optical impression.^15^

Feldspathic-, leucite- and lithium disilicate-reinforced ceramic blocks are commonly used with BRT. Lithium disilicate blocks have a high flexural strength of 360 MPa, but they require an additional sintering process after milling, which was time-consuming for single-visit treatment modalities.^17^ More translucent feldspathic- and leucite-reinforced ceramic blocks are available in monochromatic and polychromatic forms and their relatively lower fracture strength can be compensated with adhesive cementation technique.^18^ According to manufacturer’s information the flexural strength of the feldspathic ceramic blocks is 154 MPa. Their comparatively high Weibull modulus indicated a lower fracture probability compared to conventially processed feldspathic ceramics, and this is attributed to be the result of the homogeneity of the prefabricated ceramic block.^19^ The survival rate of CEREC crowns milled from feldspathic blocks has been reported to be 94.4% after 44.7±10 months.^20^

The present case report demonstrates the effective usage of CEREC 3D system with the BRT for designing and milling two central incisors in one appointment.

## Case report

A 26-year-old female patient presented herself at the Department of Prosthodontics, Istanbul University with esthetic concerns about her anterior teeth. Intraoral examination revealed that maxillary incisors restored with large composite resin restorations were aesthetically unacceptable (Figure 1A).


Figure 1. Maxillary central incisors with large composite restorations (A).Situation after removal of composite res-torations (B). Fiber posts cemented to the prepared post space with dual polymerized resin luting agent (C). Situa-tion after core build-up and tooth preparation (D).

**A:**
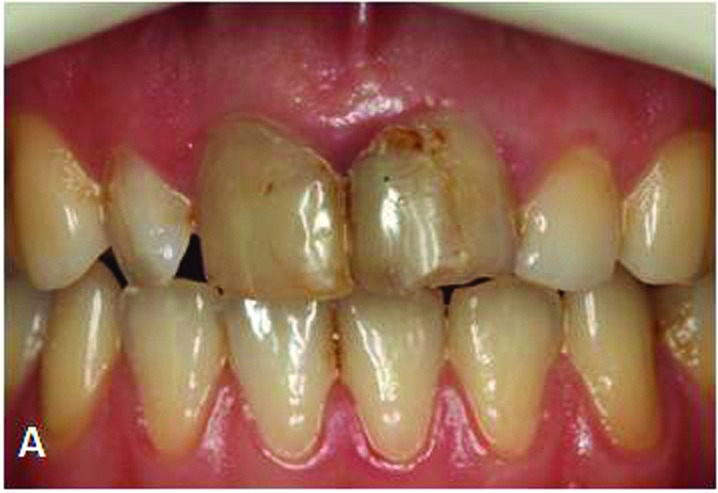

**B:**
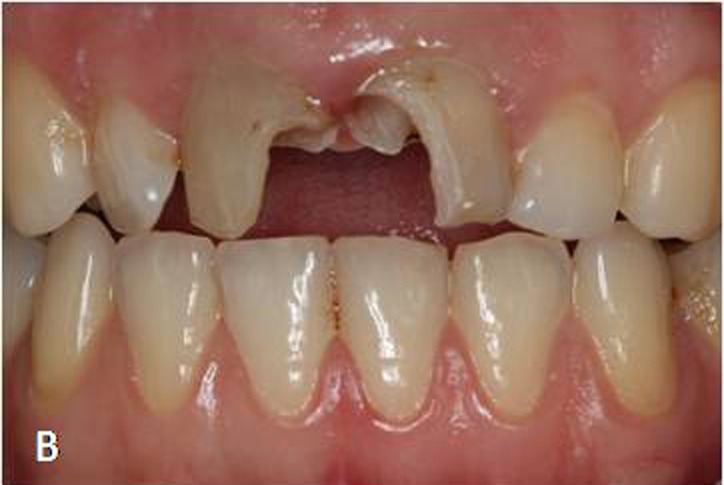

**C:**
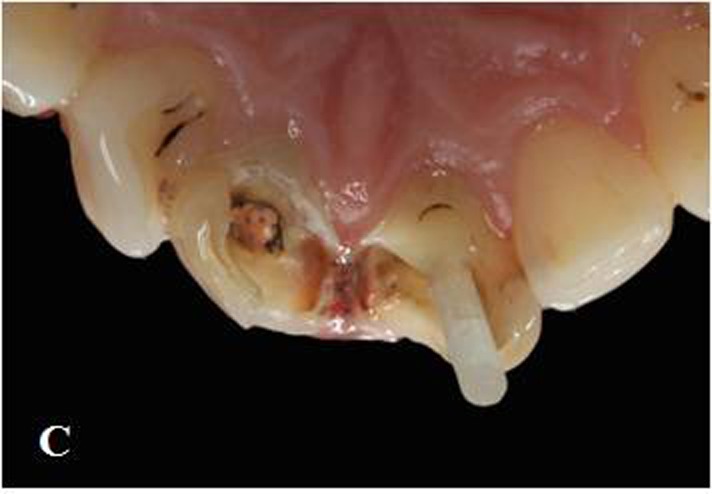

**D:**
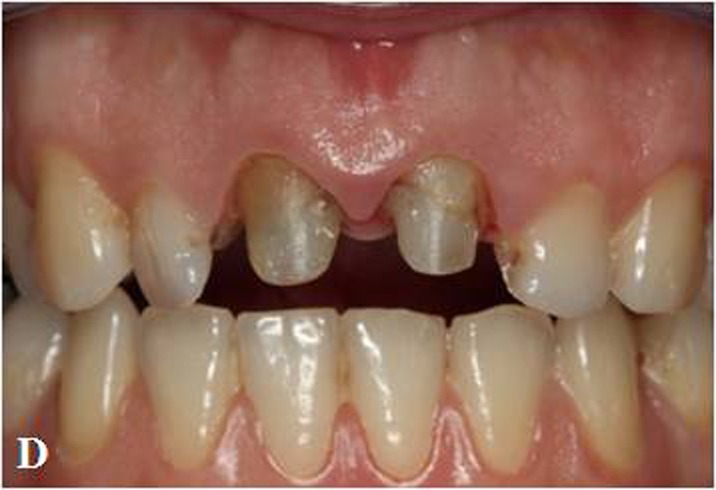

The esthetic restorative plan in this case involved fiber post and core fabrication followed by all-ceramic crowns, manufactured using the CEREC 3D system. The selection criteria for crowns designed with the regular CEREC BRT were patients with anatomically shaped contralateral teeth present. But in this case, it was aimed to represent a modified indication area of the BRT. The prosthodontic treatment plan included designing first central incisor with a conventional CAD technique, then replicating this design for the second central incisor with BRT by using the same optical impression.

Radiographic examination indicated successful endodontic treatment with a relatively wide root canal. Initially, composite resin restorations were removed to reach the root canals (Figure 1B). Gutta-percha was removed with a Peeso reamer attached to a low-speed hand-piece, and subsequently, the post preparations were made with a drill (DC3 -White-Post DC Drill; FGM, Joinville, SC, Brazil) to a depth of 8 mm. A proper-sized fiber post (#3 White Post DC; FGM, Joinville, SC, Brazil) was selected according to prepared canal dimensions and then cut at the required length.

An MDP containing self-etching primer (ED Primer II; Kuraray Medical Inc, Okayama, Japan) was used for root canal dentin conditioning because it can chemically interact with the hydroxyapatite left around the collagen within the hybrid layer and it is unaffected by the morphological variations in the post space dentin.^21,22^ A thin, uniform coat of primer was applied into the root canal with a microbrush (Microbrush X; Microbrush Corp, Grafton, Wis, USA), excess adhesive solution was absorbed with paper points and gently air-dried. Silane (Monobond S; Ivoclar Vivadent, Schaan, Liechtenstein) was applied on the post surface and waited 60 seconds for its reactions. Dual-polymerized resin cement (Clearfil Esthetic Cement; Kuraray Medical Inc, Okayama, Japan) was applied to the prepared post space with a lentulo spiral instrument, the post placed, and then photo-polymerized for 20 seconds (DEMI Led; Kerr Dental, Orange, Calif) at an intensity of 1100 mW/cm^2^ (Figure 1C).

Core build-up was made from composite resin (Clearfil Photo Core; Kuraray Medical Inc, Okayama, Japan) for preparation of abutment tooth according to manufacturer’s instructions. The tooth restored with a post-and-core system was then prepared for the planned all-ceramic restoration with subgingival chamfer margins. To provide sufficient thickness of ceramic material for strength and esthetics, circumferential 1.5 mm and incisal 2 mm tooth reductions were performed (Figure 1D). Prior to the optical impression, a retraction cord (#00 Pro Retrac; FGM, Joinville, SC, Brazil) was soaked in aluminum chloride (Hemodent; Premier Dental Products Co, Plymouth Meeting, PA) and placed in the sulcus for 5 minutes.

The designing of the restoration was started from tooth #11 by setting the CEREC 3D v.3.8 software for a crown in the biogeneric mode. Titanium-dioxide powder (CEREC Powder; Vita Zahnfabrik, Bad Säckingen, Germany) was applied to the prepared teeth and surrounding gingival tissues as an optical imaging agent. Titanium dioxide has high a refractive index and ensures uniform scattering of the light. An optical impression was made with the digital camera of the CEREC 3D acquisition unit. In this case 5 images were sufficient to capture 6 anterior teeth. Optical images of the antagonist teeth were also made and the bite registration was recorded with buccal scanning technique. In this technique optical bite registration images were taken from the buccal direction with the teeth occluded in maximum intercuspal position. In the next step, manual alignment of the preparation and antagonist models with the buccal bite registration images were required (Figure 2A). The buccal bite registration image was dragged with the mouse approximately to the corresponding parts of the preparation and antagonist models. The software then recognizes similar surfaces and automatically articulates the models in maximum intercuspal position. Once the models are virtually articulated, the occlusal contact strength of the crowns can be adjusted digitally between -200 and +200 µm, where negative values mean disclusion.


Figure 2. Optical impressions of the prepared maxillary central incisors and antagonist teeth. Digital bite regis-tration was made with buccal scanning technique. Digital bite registration (A). Determining insertion axis of the virtual crown (B). Final form of the virtual crown 11 (C). Interproximal and occlusal contact points were refined with design tools. Milling preview of the virtual crown placed in ceramic block (D). Mirroring the mor-phology of the reference tooth (E). Drawing the copy line (F).

**A:**
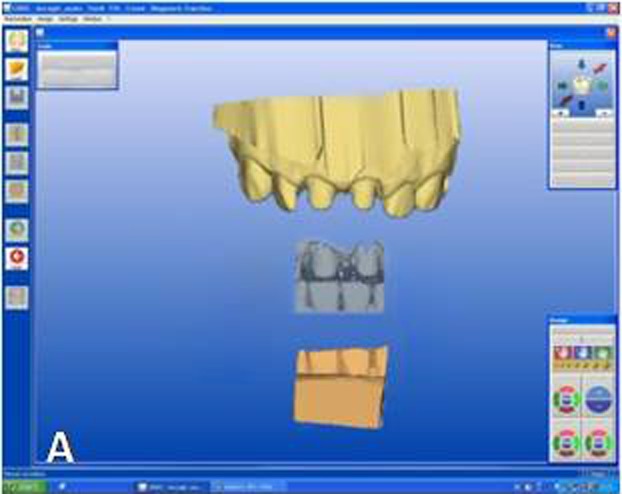

**B:**
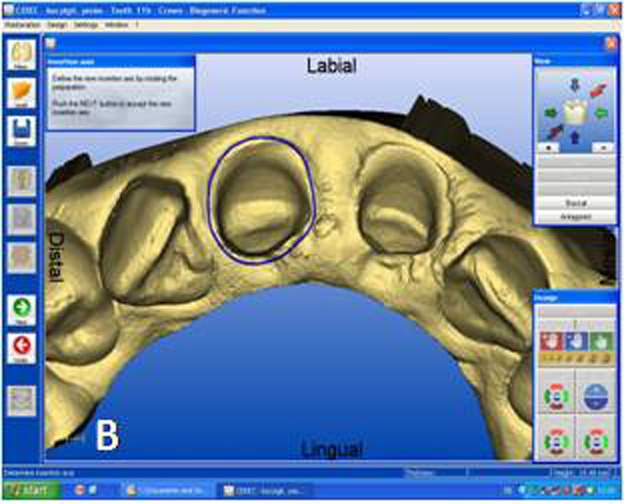

**C:**
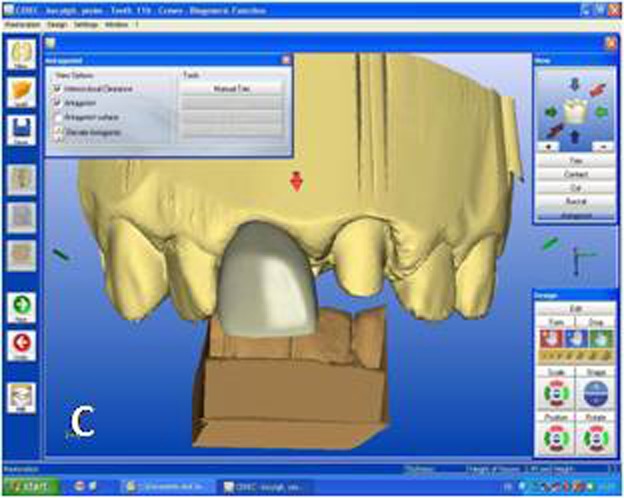

**D:**
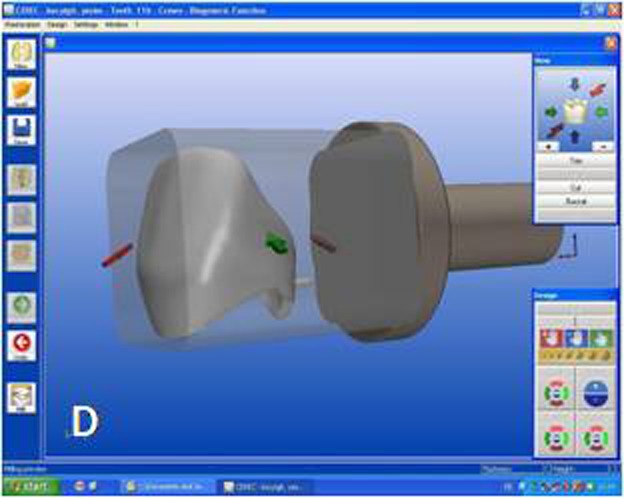

**E:**
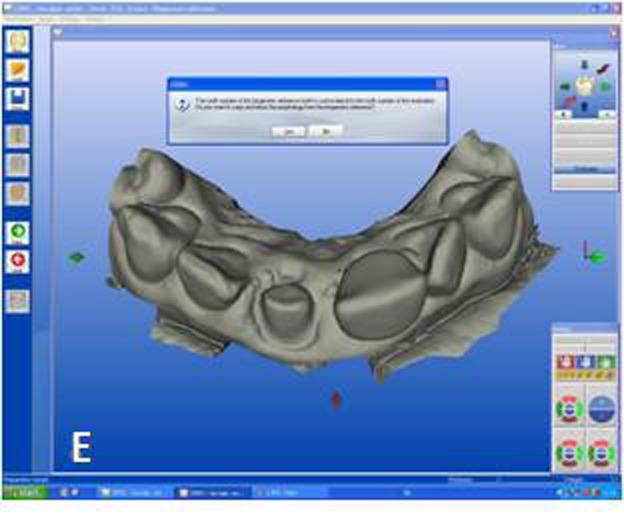

**F:**
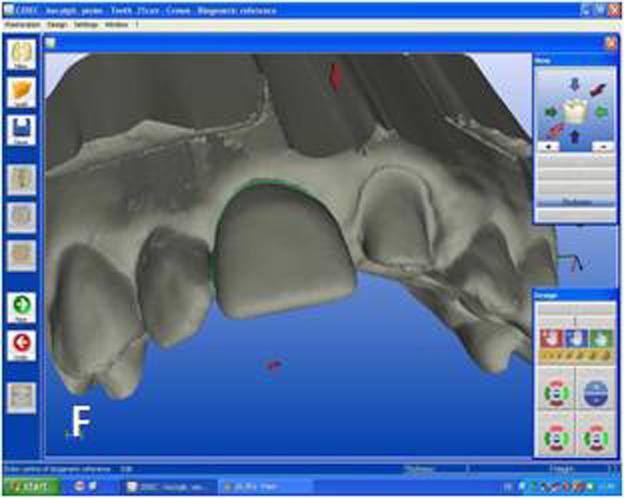

Designing the virtual restoration is similar to that of the traditionally performed at the laboratory. The first step is trimming the virtual model to attain a virtual die. Removal of neighbouring teeth in this manner reveals the interproximal margins in detail and also facilitates to shape interproximal contact points of the final restoration. Once the virtual die was approved, the preparation margins were outlined with the automatic margin finder option of the software and the insertion axis was determined (Figure 2B). CEREC software allows the operator to adjust important settings for the restoration design. Parameter settings for the present case were: proximal contact strength: 0 µm; occlusal contact strength: 0 µm; minimal thickness: 1000 µm; spacer: -30 µm. The biogeneric crown proposal was then automatically seated to the virtual die according to the adjusted settings. Interproximal and occlusal contact points were verified and the desired changes were accomplished with software’s design tools (Figure 2C). In the milling preview (Figure 2D), the restoration was placed in the feldspathic ceramic block (CEREC Blocs; Sirona Dental Systems GmbH, Bensheim, Germany) with the shade of S2M according to the CEREC Blocs shade guide.

While crown #11 was being milled, the software virtually cemented that crown, facilitating design of the next restoration. CEREC software was set in biogeneric reference mode. Trimming the virtual model, drawing the margin lines and determining the insertion axis were all made in similar fashion as for crown #11. The CEREC software prompts the practitioner to mark the tooth to be replicated. The contralateral tooth was selected and the software mirrored that tooth to make an exact copy (Figure 2E). In the next step, the desired part of the contralateral tooth was outlined to give the software the information as to which part of the tooth to copy precisely (Figure 2F). The copied tooth was aligned in the virtual model with software’s rotation and position tools that allowed manipulation of the crown in three axes (Figure 3A). After verifying interproximal and occlusal contacts, the crown was milled from the feldspathic ceramic block.


Figure 3. Final form of the virtual crown 21 (A). Final restorations after adhesive cementation (B).

**A:**
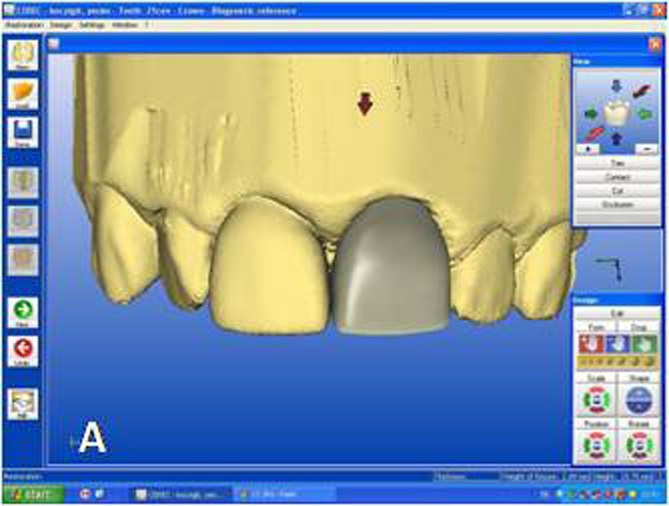

**B:**
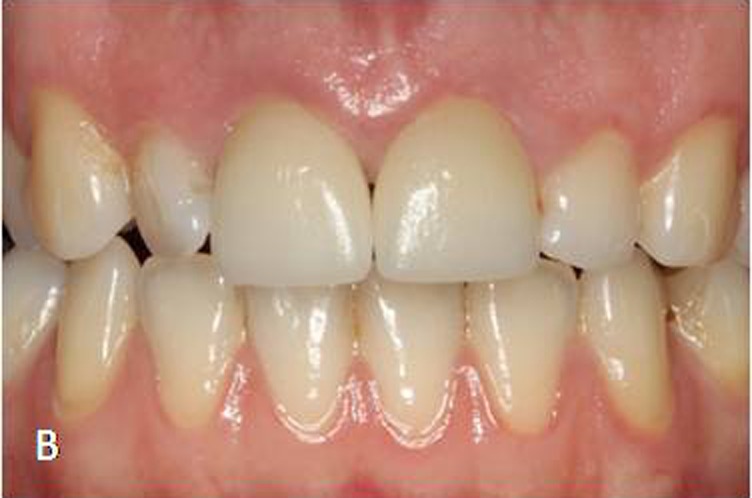

After completing try-in procedures in the patient’s mouth, custom characterization (Vita Akzent Stain and Glaze Kit; Vident, Brea, CA, USA) was accomplished and the restoration fired according to manufacturer’s specifications. The restoration was dried at 600°C for 4 minutes. Then the temperature was increased at the rate of 70°C/min for 5 minutes to 950°C and held for 1 minute. Cooling time was 3 minutes. The total glaze phase was 13 minutes.^23^

For the adhesive cementation, the ceramic crowns were etched with 5% hydrofluoric acid (IPS Empress Ceramic Etching Gel; Ivoclar Vivadent, Schaan, Liechtenstein) for 60 seconds, rinsed and air-dried. A thin layer of silane coupling agent (Monobond S; Ivoclar Vivadent, Schaan, Liechtenstein) was applied to the conditioned ceramic surfaces for 60 seconds and air-dried.^20^ For dentin conditioning, the corresponding manufacturer’s self-etching primer system (ED Primer II; Kuraray Medical Inc, Okayama, Japan) primer was used. Equal amounts of ED Primer II Liquids A and B were mixed, applied to the prepared dentin and composite core surfaces for 30 seconds and gently air-dried. The ceramic crowns were then cemented with dual-polymerized resin cement (Clearfil Esthetic Cement; Kuraray Medical Inc, Okayama, Japan). The resin cement was photo-polymerized using an LED light unit (DEMI Led; Kerr Dental, Orange, Calif) at an intensity of 1100 mW/cm² for 20 seconds each from buccal and lingual directions (Figure 3B).

## Discussion

This clinical report describes a chairside CAD-CAM technique, which enables simultaneous and symmetrical replacement of maxillary central incisors. Maxillary central incisors are the key features of an esthetic smile and should exhibit a high degree of symmetry across the midline.^24^ With conventional laboratory-fabricated crowns, this is difficult to achieve and success largely depends on the skill of the dental technician. The BRT in the CEREC 3D v.3.8 software provides the line angles and incisal edge morphology of the contralateral tooth that are exactly duplicated with great ease and speed. The quadrant feature of the software enabled the completion of two central incisors in one visit using a single optical impression.^25^ The second crown was designed while its contralateral was being milled.

Staining technique was used for color matching of milled ceramic crowns. Grinding of milled ceramic crowns for the layering ceramic can cause loss of replicated details in tooth morphology. This can adversely affect the adequate match between central incisors. In a previous study,^26^ crowns fabricated from machinable blocks were compared with the restorations obtained by an individual layering technique and it was found that layering and non-layering techniques result in little to no significant difference in esthetics.

One advantage of the technique described here is chairside color matching of ceramic restorations. One of the main problems with the laboratory-fabricated indirect ceramic restorations is communicating the hue, chroma, value, translucency, and texture with the technician. It is a time-consuming step and may require multiple visits. Digital photographs, drawings and special instructions were often used to help the dental technician understand the adjacent tooth morphology and color.^23^ With the chairside technique described here, the characterization procedures can be controlled with various colors available in the system. In case the stain does not look correct, it can be easily rinsed off and reapplied.

The patient was monitored clinically for 2 years. Ceramic fracture, surface chipping and debonding were not observed through the two-year period. The clinical success of monolithic feldspathic ceramic crowns is primarily dependent on the adhesive bonding of the resin cement to ceramic and dentin. Since dentin surface was not contaminated with temporary cements, a strong and stable dentin bonding can be achieved with single-visit chairside CAD-CAM restorations.^27^ On the other hand, fine-structured feldspathic ceramic blocks showed a highly microretentive etching pattern when etched with hydrofluoric acid, by which a strong bond to dental adhesives and luting agents is obtained.^28^ In a previous clinical study,^20^ it was concluded that adhesively cemented CEREC feldspathic ceramic crowns have provided clinical performance similar to that of ceramic core crowns. This was related to the high mechanical properties of the resin cement and the adhesion established at the interfaces between ceramic, resin cement and tooth structure.

During the follow-up period, the marginal integrity of the restoration was maintained and no gingival discoloration was observed. This may be related to the fitting accuracy of the chairside CAD-CAM restorations. Homogeneous and thin application of the light scattering powder and proper setting of luting space thickness are important factors for fitting accuracy. According to manufacturer’s report, CEREC system actually has 100 µm of internal spacer built into every restoration. Luting space parameter was set to be -30 µm (giving the value as 70 µm) before milling stage in order to compensate the imaging agent powder thickness and also achieve ideal cement gap value. May et al reported that monolithic ceramic crowns withstood higher loads when they were luted with the range of 50-100 µm cement thickness.^29^

Continued observation and addition of cases will be necessary to extend the database and provide more evidence for the reliability of the new CAD-CAM restoration techniques employing BRT technique.

## Conclusion

This case report presented restoration of maxillary central incisors using feldspathic ceramic crowns fabricated using the Biogeneric Reference Technique (BRT) mode of the CEREC 3D system. Such restorations could be accomplished in one session, without compromising the adhesion as no temporary phase is required. The described method can be especially useful for fabrication of symmetric teeth where an exact match is required.
